# Twenty-four years of response: an analysis of Global Outbreak Alert and Response Network deployments to and from the WHO Western Pacific Region

**DOI:** 10.5365/wpsar.2024.15.5.1304

**Published:** 2025-09-30

**Authors:** Sharon Salmon, Paul Effler

**Affiliations:** a World Health Organization Regional Office for the Western Pacific, Manila, Philippines.; b UNSW Medicine & Health, School of Population Health, University of New South Wales, Sydney, New South Wales, Australia.; c Pathology and Laboratory Medicine, University of Western Australia, Perth, Western Australia, Australia.

## Abstract

Since its inception in April 2000, the Global Outbreak Alert and Response Network has played a pivotal role in coordinating the rapid deployment of technical experts to support countries, when requested, during public health emergencies. This paper presents a regional analysis of the Network’s deployments within, to and from the World Health Organization Western Pacific Region over the past 24 years. The findings emphasize the critical importance of a well coordinated surge workforce and advocate for enhanced partner engagement with strategic utilization of regional and global expertise to strengthen future outbreak responses.

The Global Outbreak Alert and Response Network (GOARN) was established on 28 April 2000 as a mechanism for partners and networks around the world to provide surge support to national health authorities responding to public health emergencies. By coordinating technical expertise and resources from over 310 partners worldwide, GOARN plays an instrumental role in strengthening national responses to outbreaks of infectious diseases and other public health emergencies. GOARN’s partners include ministries of health, national public health institutes, medical and surveillance networks, academic institutions, United Nations organizations, nongovernmental organizations (NGOs) and others. GOARN’s operational coordination is provided by the Operational Support Team (OST) at the World Health Organization (WHO) headquarters and by the six WHO regional offices where technical leads work collaboratively with partners to mobilize experts and resources. (*1*-*3*)

This is the first time that GOARN’s role in supporting operational responses, both to and from the WHO Western Pacific Region, has been described and covers the period from 28 April 2000 to 31 December 2024.

## CONTEXT

GOARN comprises more than 310 partners from a wide range of institutions globally, all with a capacity to contribute resources to support countries in managing infectious disease outbreaks or public health emergencies. (*2*) When a significant outbreak or public health emergency is suspected or identified, an alert is triggered by WHO, Member States or other health partners. Alerts are typically generated for infectious diseases with a high potential for rapid spread or substantive international health risks and major natural disasters. On detection of a health emergency, the affected country, area or territory, in consultation with WHO, assesses the situation and determines the need for specialized technical assistance. Depending on the nature of the event, the expertise provided may include epidemiologists, clinicians, laboratory scientists, infection prevention and control specialists, veterinarians, communication specialists and other public health professionals. The number of experts and type of technical expertise requested vary according to the nature of the threat. Based on the assessment, a request for assistance (RFA) is drafted and circulated via the online GOARN Knowledge Platform through the designated focal point(s) from the partner institution. The RFA includes known details of the outbreak or event, the specific technical expertise required and the urgency and anticipated duration of the deployment. (*1*)

Each GOARN partner’s designated focal point is responsible for acting as a liaison between the GOARN OST and their institution. The focal point plays a key role in identifying potential experts for deployment, aligning their skills with the specific needs of the response and submitting offers of deployment through the GOARN Knowledge Platform. (*1*) Once an expert is selected, the onboarding process commences to rapidly deploy the individual to the requesting country. (*1*)

The deployment process is meticulously managed through WHO to ensure that the expert arrives with the appropriate tools, resources and knowledge to effectively respond to the public health emergency. WHO regional offices, along with country offices, facilitate the pre- and post-departure briefing of experts. Deployment duration varies depending on the nature of the response, with experts typically remaining in-country for approximately 4–6 weeks, or sometimes longer, as needed. (*4*)

GOARN partners have two primary responsibilities in supporting expert deployments. First, they are responsible for maintaining the deployed expert’s salary throughout the duration of the deployment. Second, to enable the deployed expert to concentrate fully on the response without the burden of their regular duties, partners are expected to arrange cover for the expert’s responsibilities within their home institution. (*1*)

The deployed expert collaborates closely with the WHO Incident Management Team and national health authorities, providing support for response activities in accordance with the terms of reference. These activities may include enhancing surveillance, case management, laboratory diagnostics and the implementation of effective public health interventions. (*5*)

GOARN's ability to quickly mobilize experts is crucial for controlling disease spread and minimizing the impact of health threats and provides a global mechanism for rapid collaborative responses to public health emergencies. As the outbreak or emergency is brought under control, the need for expert deployments often decreases. However, some experts may remain involved in long-term recovery efforts, such as for capacity-building, post-outbreak training and the strengthening of local health systems.

Between 2000 and 2024, GOARN facilitated the deployment of 3635 experts to 184 operations in  118 countries, areas and territories across all WHO regions, contributing approximately 121 000 person-days.

**Fig. 1:**
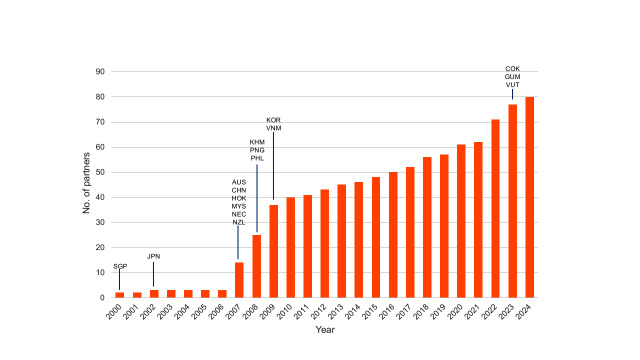
Cumulative number of GOARN partners in the WHO Western Pacific Region by year, as of 31 December 2024

The WHO Western Pacific Region, home to approximately 1.9 billion people, is spread across  37 countries and areas (before May 2025). (*6*) It is characterized by vast cultural and geographical diversity, as well as a heightened vulnerability to natural disasters and disease outbreaks. As of 31 December 2024, the WHO Western Pacific Region has  77 GOARN partners, representing colleges, governmental departments and agencies, hospitals, public health and technical institutions, networks and universities (*2*) (**Table 1**). There has been substantial growth in the number of partners since the Network’s inception (**Fig. 1**). This report describes GOARN deployments associated with the Western Pacific.

[**insert** Figure 1]

**Table 1:** List of GOARN partners in the WHO Western Pacific Region by country and area, as of 31 December 2024 (*n* = 77)

Australia (24)
1. Asia Pacific Consortium of Veterinary Epidemiology
**2. Australasian College for Infection Prevention and Control**
**3. Australian National Centre for Immunization Research and Surveillance**
**4. Burnet Institute for Medical Research and Public Health**
**5. Clinical Excellence Commission, NSW Health**
**6. Collaborative for the Advancement of Infection Prevention and Control**
**7. College of Public Health, Medical and Veterinary Sciences, James Cook University**
**8. CSIRO Australian Centre for Disease Preparedness**
**9. Doctors Without Borders – Australia**
**10. Health Emergency Branch, Department of Health and Aged Care**
**11. Hunter New England Health**
**12. Indo-Pacific Centre for Health Security, Department of Foreign Affairs and Trade**
**13. Institute for Glycomics, Griffith University, Gold Coast Campus**
**14. National Centre for Epidemiology and Population Health, The Australian National University**
**15. National Critical Care and Trauma Response Centre**
**16. PathWest Laboratory Medicine**
**17. Queensland Infection Prevention and Control Unit, Queensland Health**
**18. School of Population Health, UNSW Medicine**
**19. The Peter Doherty Institute for Infection and Immunity**
**20. The University of New South Wales, Sydney**
**21. The University of Newcastle**
**22. The University of Western Australia**
**23. University of Sydney Institute for Infectious Diseases**
**24. Westmead Hospital**

Cambodia (1)
**25. Pasteur Institute, Cambodia**

China (3)
**26. Chinese Center for Disease Control and Prevention**
**27. Guangdong Provincial Center for Disease Control and Prevention**
**28. Pasteur Institute of Shanghai, Chinese Academy of Sciences**

China, Hong Kong Special Administrative Region (3)
**29. Centre for Health Protection, Department of Health**
**30. The Chinese University of Hong Kong Special Administrative Region**
**31. University of Hong Kong Special Administrative Region**

Cook Islands (1)
**32. Public Health Directorate of the Ministry of Health**

Guam (1)
**33. Guam Department of Public Health and Social Services**

Japan (17)
**34. Association of Medical Doctors of Asia**
**35. Center for Infectious Diseases, Nara Medical University**
**36. Department of Public Health, Osaka Metropolitan University Graduate School of Medicine**
**37. Department of Virology, Tohoku University, School of Medicine**
**38. Division of Infectious Disease Prevention and Control, Ministry of Health, Labour and Welfare**
**39. Hokkaido University Research Center for Zoonosis Control**
**40. Institute of Tropical Medicine, Nagasaki University**
**41. Japan Disaster Relief Team, Japan International Cooperation Agency**
**42. Japanese Red Cross Wakayama Medical Center**
**43. Kurume University**
**44. Mie National Hospital**
**45. National Center for Global Health and Medicine**
**46. National Institute of Infectious Diseases**
**47. Osaka University**
**48. Our Lady of Snow Medical Juridical Corporation St. Mary’s Hospital**
**49. Research Institute of Nursing Care for People and Community, University of Hyogo**
**50. School of Medicine, Niigata University**

Malaysia (2)
**51. Epidemiology Intelligence Program**
**52. Institute of Health and Community Medicine, Universiti Malaysia Sarawak**

New Caledonia (2)
**53. Pasteur Institute, New Caledonia**
**54. Secretariat of the Pacific Community**

New Zealand (1)
**55. Institute of Environmental Science and Research Limited**

Papua New Guinea (2)
**56. Field Epidemiology Training Programme**
**57. Papua New Guinea Institute of Medical Research**

Philippines (3)
**58. Association of Asia Pacific Operational Research Societies**
**59. Epidemiology Bureau, Department of Health**
**60. WHO Regional Office for the Western Pacific**

Republic of Korea (3)
**61. JW LEE Center for Global Medicine, Seoul National University College of Medicine**
**62. Korea Disease Control and Prevention Agency**
**63. National Medical Centre**

Singapore (6)
**64. Ministry of Health**
**65. National Centre for Infectious Diseases**
**66. National University of Singapore**
**67. Program in Emerging Infectious Diseases, Duke-NUS Graduate Medical School**
**68. Singapore General Hospital**
**69. Tan Tock Seng Hospital**

Vanuatu (1)
**70. The Ministry of Health Surveillance, Research and Emergency Response Unit**

Viet Nam (7)
**71. Cho Ray Hospital, Ho Chi Minh City**
**72. Clinical Research Unit, Hospital for Tropical Diseases, Ha Noi**
**73. National Institute of Hygiene and Epidemiology, Ha Noi**
**74. National Institute of Malariology, Parasitology and Entomology**
**75. Pasteur Institute, Ho Chi Minh City**
**76. Pasteur Institute, Nha Trang**
**77. Tay Nguyen Institute of Hygiene and Epidemiology**

## Methods

Data were extracted from the GOARN Knowledge Platform, (*2*) an internal database that captures operational information on GOARN-supported outbreak response missions. The platform records data related to individual deployments, institutions, countries and associated technical and operational details. All outbreak response operations that triggered GOARN activation between 28 April 2000 and 31 December 2024 were included in the analysis.

Extracted variables included response operation details such as the date of activation, countries involved and the disease or event type. Deployment records were reviewed for dates of deployment, duration and technical expertise provided. Partner data included the name and type of organization (for example, academic institution, public health institute or NGO). Country-level data included the recipient country and duty station location. Where individual-level variables were missing or incomplete, such as gender, missing fields were noted but not imputed. In instances where duplicate records were identified, for example, due to overlapping deployments, manual validation was conducted to ensure accuracy and resolve inconsistencies. Data cleaning and basic descriptive analyses, including calculations of counts and medians, were performed using Microsoft Excel^®^ (Microsoft Corporation, Redmond, WA, United States of America).

## Results

**Fig. 2:**
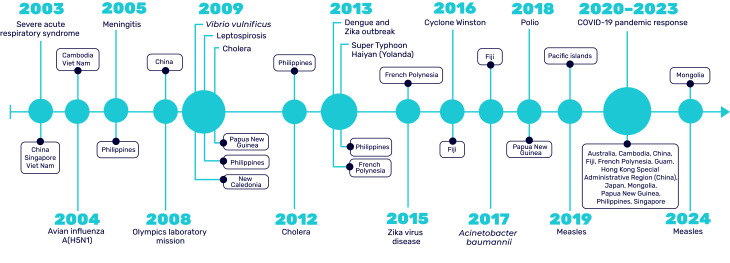
Timeline of GOARN-supported operations in the WHO Western Pacific Region, 2003–2024

Since 2003, GOARN has deployed experts almost annually to support 18 operations within the Western Pacific Region. These responses have addressed a range of infectious disease outbreaks including severe acute respiratory syndrome (SARS), avian influenza A(H5N1), meningitis, cholera, leptospirosis, *Vibrio vulnificus*, poliomyelitis, dengue, *Acinetobacter baumannii*, measles, Zika virus disease and COVID-19, (*7*) as well as emergencies triggered by natural hazards such as cyclones and typhoons (**Fig. 2**).

[**insert** Figure 2]

As of 31 December 2024, through the GOARN mechanism, 349 individuals conducted 408 missions to, from or within the WHO Western Pacific Region. The greatest number of deployments was for the COVID-19 response (*n* = 89), (*3*) followed by SARS (*n* = 78) and the West Africa Ebola outbreak (*n* = 54). Of the 349 individuals, 40 participated in more than one deployment; the greatest number of deployments by a single individual was six.

There were 106 deployments within the Western Pacific Region, 120 deployments from the Western Pacific Region to other regions and 182 deployments from other regions to the Western Pacific Region. The largest number of deployments from other regions to the Western Pacific Region was in support of the SARS response (*n* = 56) and the COVID-19 response (*n* = 47). A significant number of deployments (*n* = 54) from the Region to other regions supported the West Africa Ebola outbreak response.

A total of 25 countries, areas and territories contributed to deploying individuals through GOARN to and from the Region, including six from the Region itself. Australia contributed the highest number of experts, with 95 deployments, followed by Japan (*n* = 47) and the Philippines (*n* = 39). Notably, the deployments from the Philippines were from within the WHO Regional Office for the Western Pacific (**Table 2**).

**Table 2:** Number of individual deployments by country/area and direction of deployment, 2000–2024

Country or area	Within Western Pacific	From Western Pacific to other regions	To Western Pacific from other regions	Total
**Australia**	**55**	**40**	**–**	**95**
**Japan**	**13**	**34**	**–**	**47**
**Philippines**	**24**	**15**	**–**	**39**
**United States of Americaa**	**–**	**–**	**39**	**39**
**Switzerlanda**	**–**	**–**	**29**	**29**
**Singapore**	**10**	**16**	**–**	**26**
**Francea**	**–**	**–**	**24**	**24**
**United Kingdom of Great Britain and Northern Irelanda**	**–**	**–**	**19**	**19**
**Swedena**	**–**	**–**	**16**	**16**
**Germanya**	**–**	**–**	**14**	**14**
**Canadaa**	**–**	**–**	**13**	**13**
**Malaysia**	**1**	**11**	**–**	**12**
**Belgiuma**	**–**	**–**	**7**	**7**
**China**	**2**	**4**	**–**	**6**
**Bangladesha**	**–**	**–**	**4**	**4**
**Denmarka**	**–**	**–**	**3**	**3**
**Norwaya**	**–**	**–**	**3**	**3**
**Italya**	**–**	**–**	**2**	**2**
**Netherlandsa**	**–**	**-**	**2**	**2**
**Nigeriaa**	**–**	**–**	**2**	**2**
**Russian Federationa**	**–**	**–**	**2**	**2**
**China, Hong Kong Special Administrative Region**	**1**	**–**	**–**	**1**
**Egypta**	**–**	**–**	**1**	**1**
**Jordana**	**–**	**–**	**1**	**1**
**Spaina**	**–**	**–**	**1**	**1**
**Total**	**106**	**120**	**182**	**408**

^a^ Countries, areas or territories outside of the WHO Western Pacific Region.

Of the 41 countries that received experts, the countries from within the Western Pacific Region that received the greatest number of deployments were the Philippines (*n* = 73), followed by China (*n* = 65), Viet Nam (*n* = 63), Papua New Guinea (*n* = 42) and Fiji (*n* = 13) (**Table 3**).

**Table 3:** Global GOARN deployments by receiving country or area, 2000–2024

Receiving country or area	Within Western Pacific	From Western Pacific to other regions	To Western Pacific from other regions	Total
**Philippines**	**24**	**–**	**49**	**73**
**China**	**19**	**–**	**46**	**65**
**Viet Nam**	**18**	**–**	**45**	**63**
**Papua New Guinea**	**23**	**1**	**18**	**42**
**Sierra Leone**	**–**	**33**	**–**	**33**
**Liberia**	**–**	**20**	**–**	**20**
**Fiji**	**7**		**6**	**13**
**Bangladesh**	**–**	**12**	**–**	**12**
**Indonesia**	**–**	**12**	**–**	**12**
**Uganda**	**–**	**8**	**–**	**8**
**Cambodia**	**–**	**–**	**6**	**6**
**Singapore**	**2**	**–**	**3**	**5**
**Switzerland**	**–**	**5**	**–**	**5**
**Angola**	**–**	**4**	**–**	**4**
**French Polynesia**	**–**	**–**	**4**	**4**
**Federated States of Micronesia (Federated States of)**	**4**	**–**	**–**	**4**
**New Caledonia**	**1**	**–**	**3**	**4**
**India**	**–**	**3**	**–**	**3**
**Nigeria**	**–**	**3**	**–**	**3**
**Kiribati**	**2**	**–**	**–**	**2**
**Marshall Islands**	**2**	**–**	**–**	**2**
**Mexico**	**–**	**2**	**–**	**2**
**Mongolia**	**2**	**–**	**–**	**2**
**Sri Lanka**	**–**	**2**	**–**	**2**
**Timor-Leste**	**–**	**2**	**–**	**2**
**Tonga**	**2**	**–**	**–**	**2**
**Egypt**	**–**	**1**	**–**	**1**
**Eswatini**	**–**	**1**	**–**	**1**
**Haiti**	**–**	**1**	**–**	**1**
**Malaysia**	**–**	**–**	**1**	**1**
**Northern Mariana Islands (Commonwealth of the)**	**–**	**–**	**1**	**1**
**Pacific islands**	**–**	**1**	**–**	**1**
**Poland**	**–**	**1**	**–**	**1**
**Republic of Moldova**	**–**	**1**	**–**	**1**
**Sao Tome and Principe**	**–**	**1**	**–**	**1**
**South Africa**	**–**	**1**	**–**	**1**
**South Sudan**	**–**	**1**	**–**	**1**
**Switzerland**	**–**	**1**	**–**	**1**
**United Arab Emirates**	**–**	**1**	**–**	**1**
**Yemen**	**–**	**1**	**–**	**1**
**Zimbabwe**	**–**	**1**	**–**	**1**
**Total**	**106**	**120**	**182**	**408**

A total of 64 partners were involved in the 408 Region-associated deployments. GOARN partners that deployed the greatest number of individuals included the WHO Regional Office for the Western Pacific (*n* = 39), the United States Centers for Disease Control and Prevention (*n* = 38) and the Australian Response MAE (Master of Applied Epidemiology) Network (*n* = 30) (**Table 4**).

**Table 4:** Number of individuals deployed to, from or within the Western Pacific Region by GOARN partner, 2000–2024

Deploying GOARN partner	No. of individuals
**WHO Regional Office for the Western Pacific**	**39**
**United States Centers for Disease Control and Prevention**	**38**
**ARM Network (Australian Response MAE Network)**	**30**
**National Institute of Infectious Diseases, Japan**	**26**
**GOARN Secretariat, Switzerland**	**24**
**National Centre for Epidemiology and Population Health, The Australian National University**	**15**
**Division of Tuberculosis and Infectious Disease Control, Ministry of Health, Labour and Welfare, Japan**	**14**
**National University of Singapore**	**14**
**Robert Koch Institute, Germany**	**14**
**Public Health Agency of Canada**	**13**
**Hunter New England Health, Australia**	**12**
**Santé publique, France**	**12**
**Institute of Health and Community Medicine, Universiti Malaysia Sarawak, Malaysia**	**10**
**United Kingdom Health Security Agency, United Kingdom of Great Britain and Northern Ireland**	**10**
**Burnet Institute for Medical Research and Public Health, Australia**	**9**
**United Kingdom Public Health Rapid Support Team, United Kingdom of Great Britain and Northern Ireland**	**8**
**European Centre for Disease Prevention and Control**	**7**
**Chinese Center for Disease Control and Prevention**	**6**
**European Programme for Intervention Epidemiology Training**	**6**
**Médecins Sans Frontières – International Office**	**6**
**Ministry of Health, Singapore**	**6**
**Australasian College for Infection Prevention and Control**	**5**
**EPIET Alumni Network**	**5**
**Faculty of Health Sciences, Curtin University of Technology, Australia**	**5**
**International Centre for Diarrhoeal Disease Research, Bangladesh**	**4**
**National Center for Global Health and Medicine, Japan**	**4**
**National Centre for Infectious Diseases, Singapore**	**4**
**The University of Newcastle, Australia**	**4**
**Department of Virology, Tohoku University School of Medicine, Japan**	**3**
**Doherty Institute, Australia**	**3**
**EPITER: Association pour le développement de l'épidémiologie de terrain, France**	**3**
**Norwegian Institute of Public Health**	**3**
**Public Health Agency of Sweden**	**3**
**WHO WHE Operations Support and Logistics**	**3**
**Epidemiology Intelligence Program, Malaysia**	**2**
**Health Emergency Branch, Department of Health and Aged Care, Australia**	**2**
**Indo-Pacific Centre for Health Security, Department of Foreign Affairs and Trade, Australia**	**2**
**National Critical Care and Trauma Response Centre, Australia**	**2**
**Pasteur Institute, Cambodia**	**2**
**Rospotrebnadzor, Russian Federation**	**2**
**The University of New South Wales, Australia**	**2**
**Westmead Hospital, Australia**	**2**
**WHO Regional Office for Europe**	**2**
**World Organization for Animal Health**	**2**
**Association of Medical Doctors of Asia, Japan**	**1**
**Australian National Centre for Immunization Research and Surveillance**	**1**
**Eastern Mediterranean Public Health Network**	**1**
**Emerging and Dangerous Pathogens Laboratory Network**	**1**
**Erasmus University Medical Center, Netherlands**	**1**
**Federal University Oye Ekiti, Nigeria**	**1**
**Global Infection Prevention and Control Network**	**1**
**Instituto de Salud Carlos III, Spain**	**1**
**Istituto Superiore di Sanita, Italy**	**1**
**London School of Hygiene and Tropical Medicine, United Kingdom of Great Britain and Northern Ireland**	**1**
**Médecins Sans Frontières, Belgium**	**1**
**National Institute for Public Health and the Environment, Netherlands**	**1**
**Nigeria Centre for Disease Control, Nigeria**	**1**
**Queensland Infection Prevention and Control Unit, Australia**	**1**
**Statens Serum Institut, Denmark**	**1**
**Tan Tock Seng Hospital, Singapore**	**1**
**United Nations Food and Agriculture Organization**	**1**
**University of Hong Kong Special Administrative Region, Hong Kong Special Administrative Region, China**	**1**
**University of Nebraska Medical Center, United States of America**	**1**
**WHO Regional Office for the Eastern Mediterranean**	**1**
**Total**	**408**

GOARN: Global Outbreak Alert and Response Network; MAE: Master of Applied Epidemiology; WHE: WHO Health Emergencies Programme; WHO: World Health Organization.

**Fig. 3:**
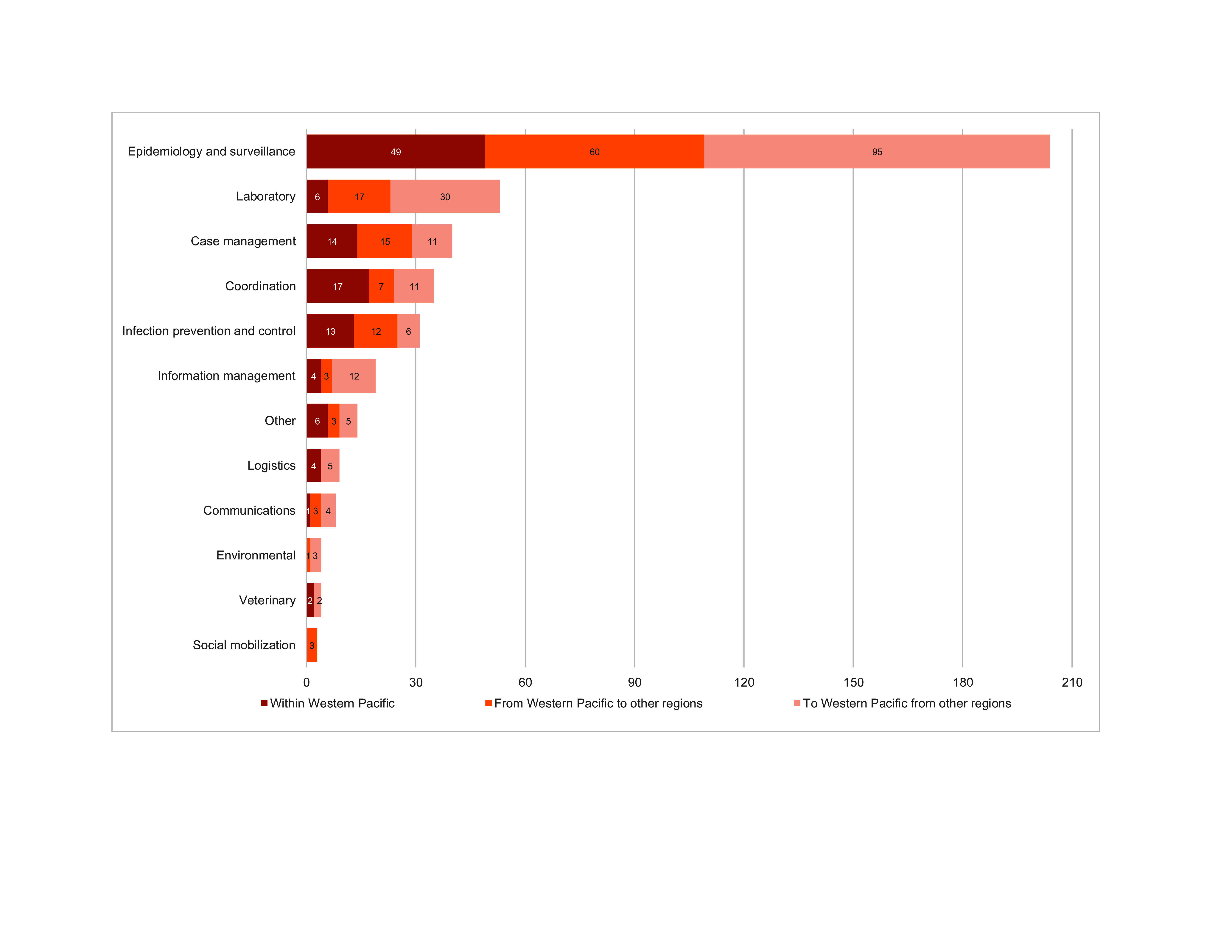
Number and type of technical expertise deployed by GOARN partners by geographical location, as of 31 December 2024

Of the 408 deployments, the most requested technical expertise was epidemiology and surveillance (*n* = 204), followed by technical laboratory assistance (*n* = 53). The number and type of technical expertise deployed by GOARN partners by geographic location is shown in **Fig. 3**.

[**insert** Figure 3]

GOARN experts supported 40 operations ranging from infectious disease outbreaks to natural disasters. Of the 408 deployments, 106 (26%) were deployed within the Western Pacific Region, 120 (30%) were deployed from the Western Pacific Region to other regions, and 182 (45%) came from other regions to support responses within the Western Pacific Region (**Table 5**).

**Table 5:** Number of individuals deployed by operation involving the Western Pacific Region, categorized by deployment origin, as of 31 December 2024

Year	Operation	Within Western Pacific	Western Pacific to other regions	Other regions to Western Pacific	Total
**2000**	**Ebola haemorrhagic fever, Uganda**	**–**	**8**	**–**	**8**
**2003**	**Severe acute respiratory syndrome, China, Singapore, Viet Nam**	**22**	**–**	**56**	**78**
**2004**	**Avian influenza A(H5N1), Viet Nam**	**11**	**–**	**19**	**30**
**2004**	**Tsunami, India, Indonesia**	**–**	**11**	**–**	**11**
**2004**	**Avian influenza A(H5N1), Cambodia**	**–**	**–**	**3**	**3**
**2004**	**Avian influenza A(H5N1), Indonesia**	**–**	**2**	**–**	**2**
**2004**	**Nipah, Bangladesh**	**–**	**2**	**–**	**2**
**2005**	**Meningitis, Philippines**	**3**	**–**	**6**	**9**
**2005**	**Marburg virus international outbreak response, Angola**	**–**	**4**	**–**	**4**
**2005**	**Dengue haemorrhagic fever, Timor-Leste**	**–**	**2**	**–**	**2**
**2005**	**Myocarditis, Sri Lanka**	**–**	**2**	**–**	**2**
**2006**	**Avian influenza: coordination of response operations, Indonesia**	**–**	**1**	**–**	**1**
**2008**	**Olympics laboratory mission, China**	**1**	**–**	**3**	**4**
**2008**	**Cholera and anthrax outbreak, Zimbabwe**	**–**	**1**	**–**	**1**
**2009**	**Cholera outbreak, Papua New Guinea**	**1**	**–**	**8**	**9**
**2009**	**Pandemic influenza A(H1N1) investigation and response, multicountry**	**–**	**6**	**–**	**6**
**2009**	**Leptospirosis outbreak, Philippines**	**2**	**–**	**3**	**5**
**2009**	***Vibrio vulnificus*, New Caledonia**	**1**	**–**	**3**	**4**
**2010**	**Cholera outbreak, Haiti**	**–**	**1**	**–**	**1**
**2012**	**Cholera outbreak, Philippines**	**–**	**–**	**2**	**2**
**2013**	**Disaster and conflict response mission, Philippines**	**13**	**–**	**24**	**37**
**2013**	**Dengue and Zika outbreak, French Polynesia**	**–**	**–**	**1**	**1**
**2013**	**Middle East respiratory syndrome, ** **Eastern Mediterranean**	**–**	**1**	**–**	**1**
**2014**	**Ebola outbreak, West Africa**	**–**	**54**	**–**	**54**
**2014**	**Cholera outbreak, South Sudan**	**–**	**1**	**–**	**1**
**2015**	**Zika virus disease, multiple locations**	**–**	**–**	**2**	**2**
**2016**	**Cyclone Winston, Fiji**	**5**	**–**	**1**	**6**
**2017**	**Rohingya crisis (protracted emergency), Bangladesh**	**–**	**10**	**–**	**10**
**2017**	**Acinetobacter baumannii, Fiji**	**2**	**–**	**1**	**3**
**2018**	**Poliomyelitis, Papua New Guinea**	**2**	**1**	**1**	**4**
**2018**	**Lassa fever, Nigeria**	**–**	**2**	**–**	**2**
**2018**	**Ebola virus disease preparedness and readiness, Democratic Republic of the Congo**	**–**	**1**	**–**	**1**
**2018**	**Listeriosis, South Africa**	**–**	**1**	**–**	**1**
**2019**	**Measles, Pacific island countries and areas**	**2**	**1**	**2**	**5**
**2019**	**Cholera outbreak, Yemen**	**–**	**1**	**–**	**1**
**2019**	**Lassa fever, Nigeria**	**–**	**1**	**–**	**1**
**2021**	**COVID-19 global response**	**39**	**2**	**47**	**88**
**2021**	**COVID-19 vaccination response, ** **African Region**	**–**	**1**	**–**	**1**
**2022**	**Ukraine emergency**	**–**	**2**	**–**	**2**
**2022**	**Dengue fever, Sao Tome and Principe**	**–**	**1**	**–**	**1**
**2024**	**Measles, Mongolia**	**2**	**–**	**–**	**2**
	**Total**	**106**	**120**	**182**	**408**

The median length of deployment was 27 days, with half of all assignments ranging between 16 and 42 days. The COVID-19 pandemic operation recorded the longest deployments, with one expert deployed to Papua New Guinea for 191 days, followed by the Philippines for 133 days and Fiji for 121 days. In addition, one expert provided remote technical support to an in-person team during this period. (*4*)

## Discussion

The findings emphasize GOARN’s critical role in strengthening outbreak response capabilities through timely technical surge support. Deployments addressed a wide array of public health emergencies from emerging infectious diseases such as SARS, Zika and COVID-19 to recurrent threats like dengue and measles. This breadth of activity highlights the adaptability and reach of the GOARN mechanism in supporting not only acute responses but also, where needed, post-emergency recovery and capacity-building efforts.

The involvement of Western Pacific Region partners in deployments beyond their own region highlights GOARN’s dual function as both a responder to local emergencies and a contributor to global health security. Countries, such as Australia and Japan, have consistently supported international operations, underscoring the value of cross-regional collaboration and shared responsibility in addressing global public health threats.

The predominance of deployments for epidemiology and surveillance assistance reflects the need for timely detection, case investigation and outbreak monitoring. However, the comparatively lower number of deployments in other important areas, such as risk communication, logistics and coordination, suggests opportunities to further strengthen multidisciplinary surge capacities across the Network. The recurring involvement of key institutions – such as the WHO Regional Office for the Western Pacific, the United States Centers for Disease Control and Prevention and the Australian Response MAE Network – demonstrates the importance of sustained engagement by a core group of active and reliable partners. Continued investment in the readiness of all GOARN partners, especially those less frequently involved, could help expand the pool of deployable expertise and enhance the Network’s flexibility.

Operationally, several challenges persist, including aligning expert availability with the rapidly changing demands of outbreak contexts, navigating complex cross-border logistical requirements and sustaining voluntary contributions from partner institutions. GOARN’s model, which relies on partners to maintain deployed staff salaries and backfill their roles, has been key to its function but raises questions about sustainability, especially during large-scale or prolonged responses. Strengthening institutional agreements, investing in rapid deployment mechanisms and leveraging resources, such as WHO collaborating centres, may help improve the predictability and scalability of future mobilizations.

The lack of gender-disaggregated data limits any analysis of equity in deployment participation. As gender equity becomes an increasingly important component of the global health workforce agenda, future data systems should include demographic indicators to support more inclusive monitoring and reporting. This will be essential for fostering greater diversity, ensuring equal participation and promoting equity within GOARN’s deployments.

### Limitations

This analysis has several limitations. First, the completeness and consistency of deployment records may vary over time, particularly in the earlier years of GOARN’s operations, potentially affecting the accuracy of trend analysis. The analysis does not assess the direct impact of deployments; however, there are published reports on health outcomes and system capacity strengthening. (*8*) Contextual factors influencing deployments, such as political or logistical constraints, were not systematically captured. Additionally, gender-disaggregated data were not available, limiting the ability to assess gender representation among deployed experts. Finally, informal or undocumented contributions may not be reflected, leading to the potential underestimation of the full scope of GOARN’s utilization in the Region.

### Conclusion

Over the past 24 years, GOARN’s work in the WHO Western Pacific Region has exemplified the power of coordinated international collaboration in responding to infectious disease outbreaks. The analysis showed that GOARN has successfully established a vast international infrastructure of partners, capable of rapid, collaborative action during public health emergencies. This infrastructure has proven highly effective – demonstrated by the number, diversity and growth of deployments, alongside an exponential increase in participating partners over time.

As a core partner of the Global Health Emergency Corps, GOARN plays a pivotal role in shaping a more agile, interoperable and prepared global response architecture. Its experience in the Region offers valuable lessons for strengthening global emergency preparedness, underscoring the importance of trusted partnerships, standardized mechanisms and strong technical coordination.

In an increasingly interconnected world, GOARN is well positioned to continue enabling timely, effective and equitable outbreak responses across regions.
